# TaGRF3-2A Improves Some Agronomically Valuable Traits in Semi-Dwarf Spring Triticale

**DOI:** 10.3390/plants10102012

**Published:** 2021-09-25

**Authors:** Mikhail Divashuk, Anastasiya Chernook, Aleksandra Kroupina, Milena Vukovic, Gennady Karlov, Aleksey Ermolaev, Sergey Shirnin, Sergey Avdeev, Vladimir Igonin, Vladimir Pylnev, Pavel Kroupin

**Affiliations:** 1 All-Russia Research Institute of Agricultural Biotechnology, Timiryazevskaya Street, 42, 127550 Moscow, Russia; irbis-sibri@yandex.ru (A.C.); annshirley@yandex.ru (A.K.); milenna.vukovic@gmail.com (M.V.); karlov@iab.ac.ru (G.K.); ermol-2012@yandex.ru (A.E.); durandal-1707@bk.ru (S.S.); selection@timacad.ru (V.I.); pavelkroupin1985@gmail.com (P.K.); 2 Institute of Agrobiotechnology, Russian State Agrarian University—Moscow Timiryazev Agricultural Academy, Timiryazevskaya Street, 49, 127550 Moscow, Russia; avdeev@rgau-msha.ru (S.A.); pyl8@yandex.ru (V.P.)

**Keywords:** triticale, *TaGRF3*-2A, *DDW1*, yield, molecular marker

## Abstract

The breeding improvement of triticale is tightly associated with the introgression of dwarfing genes, in particular, gibberellin (GA)-insensitive *Ddw1* from rye. Despite the increase in harvest index and resistance to lodging, this gene adversely affects grain weight and size. Growth regulation factor (*GRF*) genes are plant-specific transcription factors that play an important role in plant growth, including GA-induced stem elongation. This study presents the results of a two-year field experiment to assess the effect of alleles of the *TaGRF3*-2A gene in interaction with *DDW1* on economically valuable traits of spring triticale plants grown in the Non-Chernozem zone. Our results show that, depending on the allelic state, the *TaGRF3*-2A gene in semi-dwarf spring triticale plants influences the thousand grain weight and the grain weight of the main spike in spring triticale, which makes it possible to use it to compensate for the negative effects of the dwarfing allele *Ddw1*. The identified allelic variants of the *TaGRF3*-2A gene can be included in marker-assisted breeding for triticale to improve traits.

## 1. Introduction

Triticale is an important food crop that combines the genomes of wheat and rye. Due to its high ecological plasticity and adaptability, triticale is successfully grown in poor soils and is resistant to a wide range of fungal diseases [1,2,3,4,5,6,7,8]. Triticale is grown as a forage crop for grain and green mass. Recently, triticale grain has also been used for the production of biogas and bioethanol, and it may also have prospects in the production of functional foods [9,10,11].

Triticale breeding has made significant progress; thanks to breeding, it was possible to improve its feeding qualities and remove such undesirable traits as preharvest sprouting and lodging. As a result of lodging, the straw cannot withstand a heavy ear and bends towards the ground. Lodging reduces the efficiency of photosynthesis due to changes in the architecture of the stem, leads to seed germination and phytotoxin contamination, slows down maturation, and requires additional costs for drying [12,13].

The development of semi-dwarf varieties is a reliable way to solve the lodging problem. Dwarfing genes reduce the height of the straw, increasing the yield via higher grain number per ear yield without the risk of losing it as a result of lodging [14,15]. However, in experiments with isogenic recombinant lines, the dwarfing genes, as a rule, reduce the thousand grain weight, impair the assimilation of soil nitrate nitrogen, and reduce drought resistance, and they can extend the periods of flowering and heading [16,17].

Growth regulation factor (*Grf*) genes are plant-specific transcription factors that play an important role in plant growth, including gibberellin (GA)-induced stem elongation [18]. In rice, a mechanism for the regulation of the activity of DELLA protein, which inhibits nitrogen assimilation and carbon fixation, by means of OsGRF4 was revealed: in dwarf rice plants and transgenic wheat plants with the GRF4^ngr2^ allele, the activities of glutamate synthase and nitrate reductase were higher than those in control plants. As a result of the expression of this allele, a larger grain was formed while maintaining the semi-dwarf phenotype [19,20]. In common wheat, 30 *TaGRF* genes were identified on 12 chromosomes, which were divided into four phylogenetic groups [21,22]. Analysis of the *TaGRF3* gene sequence on the 2D common wheat chromosome using molecular markers showed its allelic diversity among the accessions of the collection, as well as a correlation with thousand grain weight and the size of wheat kernels [23]. A number of authors propose that it is the tuning of genes of nitrogen metabolism in low-stemmed varieties that will drive a new wave of the green revolution, increasing the absorption of nitrogen and, as a consequence, the weight of the grain [19,24,25].

The triticale genome makes it possible to successfully combine genes for dwarfism from both wheat and rye. Among the dwarfing genes of rye, the dominant allele *Ddw1* has the greatest agricultural value. The Gibberellin 2-Oxidase gene is considered a candidate for *Ddw1*, while its ortholog in wheat is considered to be the *Rht12* gene [14,26,27,28,29,30]. It is able to reduce the height of plants by 25–30%, but at the same time, it reduces the thousand grain weight and the weight of grains per ear [31,32,33,34,35,36,37]. *Ddw1* is successfully used in triticale breeding in Poland, Romania, and other European countries [14,38,39]. In our work, we carried out a two-year study on the interaction of the *TaGRF3-2A* (hereafter designated in the test as *Grf3*) and *Ddw1* genes in spring triticale in the field conditions of the Central Non-Chernozem (Non-Black Earth) Region of the Russian Federation.

## 2. Results

The field experiments in 2018 and 2019 differed in terms of meteorological conditions (Table 1). In general, 2019 was more favorable for the formation of the spring triticale yield for all families and any combination of genes. The yield elements formed in 2019, on average, were higher than those in 2018: the vegetative mass of the main spike in 2019 exceeded that in 2018 by 17% (0.5 g), the grain weight of the main spike was increased by 18% (0.4 g), the thousand grain weight was increased by 22% (11.3 g), the number of fertile tillers was on average 33% higher (one shoot), and the harvesting index was 8% higher. In addition, in 2018, only 23.5% of plants had six internodes; in 2019, 41.3% of plants had six internodes.

The effect of *Ddw1* on economically valuable traits of spring triticale in our experiments generally showed trends the same as those in studies by other authors and in our previous studies [31,32,33,34,35,40]. On average, for both years of the experiment, *Ddw1* reduced the height of spring triticale plants by 33% (28.2 cm). The vegetative mass of the main spike and the grain weight of the main spike, on average, in semi-dwarf plants (*Ddw1Ddw1*) were lower than those in tall plants (*ddw1ddw1*) by 10% (0.3 g) and 9% (0.2 g), respectively. The thousand grain weight in semi-dwarf plants was, on average, 6% lower, and the presence of *Ddw1*, on average, increased the harvesting index by 8% (0.05 units). Plants of spring triticale with *Ddw1*, on average, came to heading and anthesis 5 days later than did tall triticale plants.

### 2.1. Spike Architecture and Productivity

We revealed a statistically significant effect of allelic variants of the *TaGRF3-2A* (hereafter designated in the test as *Grf3*) gene upon interaction with the *Ddw1* gene on the grain weight of the main spike in both generations.

**Figure 1:**
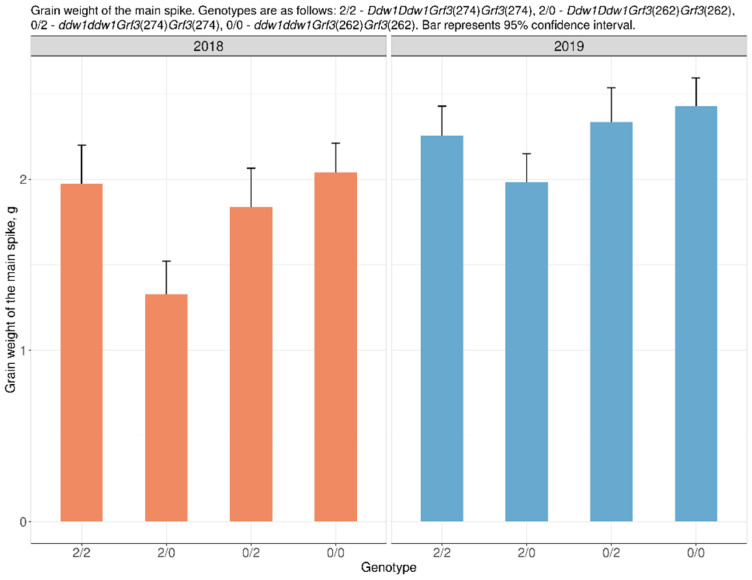
The effect of the allelic state of *DDW1* and *Grf3* on grain weight of the main spike.

The grain weight of the main spike in F_4:5_ (2018) in semi-dwarf *Ddw1*-carrying plants with the *Grf3*(274) allele was 35% (0.7 g) higher than that in such plants with the *Grf3*(262) allele. In F_5:6_ (2019), the *Grf3*(274) allele increased the grain weight from the main spike by 12% (0.3 g) (Table S1) (Figure 1). In tall plants of spring triticale (without *Ddw1*) in F_4:5_ (2018) and F_5:6_ (2019), no statistically significant difference was observed in the grain weight per spike between different *Grf3* genotypes.

**Figure 2:**
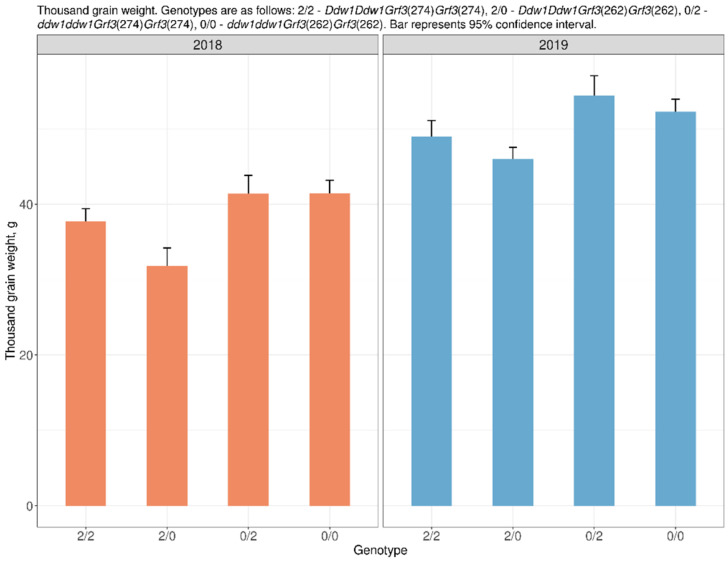
The effect of the allelic state of *DDW1* and *Grf3* on thousand grain weight.

The thousand grain weight in spring triticale plants with the dwarfing *Ddw1* and *Grf3*(274) alleles was statistically significantly higher by 5.94 g (16%) compared to that in semi-dwarf plants with the *Ddw1*/*Grf3*(262) combination in F_4:5_ (2018). In F_5:6_ (2019), the difference between the same combinations of alleles was 2.96 g (6%), but no statistical significance was found. In tall plants of spring triticale (without *Ddw1*) in F_4:5_ (2018) and F_5:6_ (2019), no statistically significant difference in thousand grain weight between different allelic variants of the *Grf3* gene was found. The number of grains per main spike did not show statistically significant differences between the combinations of genes in both years of the study, although certain trends were observed (Table S1, Figure 2).

### 2.2. Flowering and Heading

**Figure 3:**
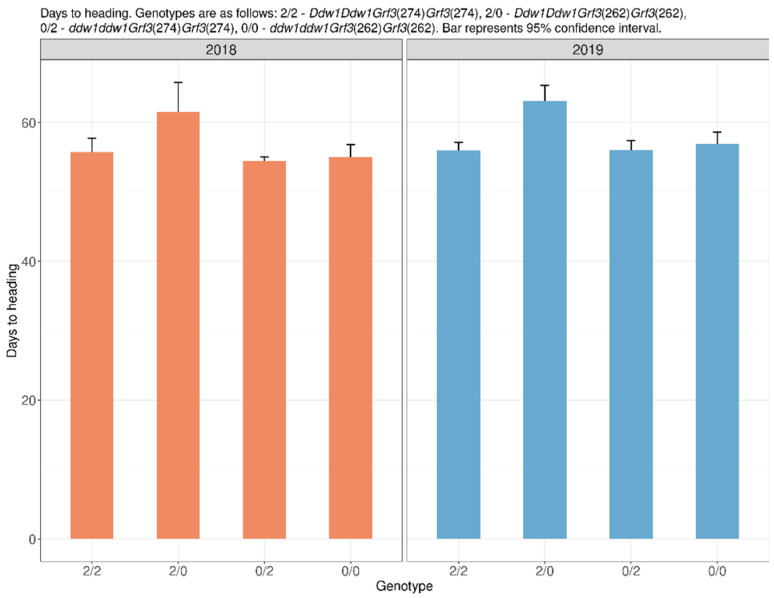
The effect of the allelic state of *DDW1* and *Grf3* on period duration from sowing to heading.

We obtained a rather unexpected effect when assessing the effect of the combination of the studied genes on flowering and heading. As expected, the presence of *Ddw1* resulted in later flowering: semi-dwarf plants bloomed, on average, 5 days later than tall plants. However, when taking into account the allelic state of the *Grf3* gene, it was found that in F_5:6_ (2019) in semi-dwarf plants with the *Grf3*(274) allele, heading and anthesis occurred, on average, 7 days earlier than in plants with the *Grf3*(262). In F_4:5_ (2018), the difference was 5–6 days, but due to unfavorable conditions in 2018, the transition between the phases was significantly extended in time, which did not result in statistically significant differences. In tall plants, the *Grf3* gene had no effect on the heading and anthesis time (Table S1, Figure 3).

### 2.3. Plant Height, Internodes, and Main Spike Length

**Figure 4:**
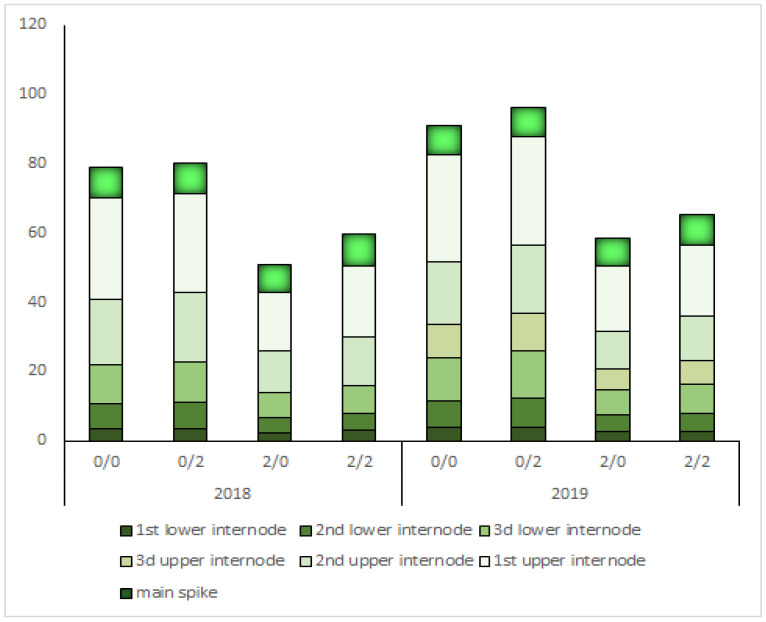
Schematic diagram showing the internode and spike length of the main shoot of spring triticale plants with different combinations of the *DDW1* and *Grf3* alleles. Genotypes are as follows: 2/2 *Ddw1Ddw1Grf3*(274)*Grf3*(274), 2/0 *Ddw1Ddw1Grf3*(262)*Grf3*(262), 0/2 *ddw1ddw1Grf3*(274)*Grf3*(274), *0/0 ddw1ddw1Grf3*(262)*Grf3*(262).

An analysis of the effect of *Ddw1* on the anatomical structure of the stem in the field experiment showed the expected results of a significant decrease in plant height (Table S1, Figure 4).

The allelic state *Grf3* had little effect on plant height. Allele *Grf3*(274) insignificantly increased plant height in all combinations by from 1 to 6 cm. However, this was more obvious in semi-dwarf plants. The plants with the *Grf3*(274) allele were 6 cm taller than those with the *Grf3*(262) allele.

## 3. Discussion

Experimental conditions in the Non-Black Earth Zone in 2018 and 2019 differed both in meteorological conditions and in average plant productivity. Thus, in the present study, we compared two different years, which allowed us to compare the manifestation of *TaGRF3-2A* (designated here as *Grf3*) under different conditions in low-stem and high-stem plants of spring triticale.

In the present study, we showed that the allelic state of *TaGRF3-2A* in semi-dwarf plants carrying *Ddw1* affects plant productivity, thousand grain weight, and grain weight from the main spike, demonstrating a partial compensatory effect on these traits in comparison with the negative effect of the *Ddw1* gene. At the same time, it can be noted that the effect manifested more significantly under 2018 conditions, which were unfavorable for spring triticale. In our previous study [34], we showed the effect of *TaGRF3-2D* on the weight and dimensions of the seed in a bread wheat collection. Zan et al. (2020) showed that the *TaGRF3* gene (as well as its B- and D-genome homologues *TaGRF-15* and *TaGRF-23*) has an increased level of expression in the ear [41].

The effect of *Ddw1* on the linear parameters of the stem under the influence in both years of the field experience was quite similar: the overall decrease was 33%, which is generally consistent with previous studies [31,32,33,34,35]. On the whole, *TaGRF3-2A* did not have a significant effect on the height of triticale plants; however, the allele state *Grf3*(274) increased the length of individual internodes and the length of the spike. Expression data from Zan (2020) and Huang (2021) showed that *TaGRF3*-2A (*Grf3*) has higher expression in the stem and apical meristem. Thus, the presence of the *TaGRF3-2A* (274) allele in semi-dwarf plants leads to only a slight increase in plant height (up to 6 cm) and, therefore, does not interfere with the semi-dwarf phenotype of the variety required for intensive cultivation technology and control of lodging [22,41].

It is known that both genes studied by us are involved in gibberellin pathways and thus affect the linear dimensions of the stem, ear, and grain. The gibberellin-sensitive *Ddw1* gene was shown to co-segregate with the *ScGA2ox12* gibberellin-2 oxidase gene [30], while its most likely wheat homolog, the *Rht12* gene, probably decreases plant height through its influence on the *TaGA2ox-A14* gibberellin metabolism gene [42,43]. Rice *OsGRF* genes, through participation in gibberellin pathways and in interaction with miR396, affect stem length [18], grain size [20], and plant architecture as a whole [44]. Thus, we can assume the interaction of *TaGRF3-2A* and DELLA proteins in the formation of the stem and spike such as at the stage of the apical meristem, the initiation of generative organs in the spike, stem growth, and grain filling by means of gibberellin pathways.

It is known that gibberellin-sensitive dwarfing genes significantly extend the periods of heading and anthesis in cereals. This has been demonstrated for the *Ddw1* genes in triticale [32,33,34,35,45,46], which negatively correlate with Fusarium spike resistance. A similar effect was also noted in its most likely homologue, the *Rht12* gene in wheat [47,48,49]. The later the flowering, the shorter the favorable period for grain filling [50]. We showed that due to the presence of *TaGRF3-2A* (274) in semi-dwarf plants, flowering and heading occur earlier, which increases the period for grain formation. As a discussion, we can assume the hormonal influence of the *TaGRF3-2A* (274) allele on the rate of cell division/elongation in grain, since we have shown its positive effect on an increase in the linear dimensions of the ear and stem. However, the study of the influence of the *TaGRF3*-2A gene requires further field studies in different crops and molecular genetic studies to study its mechanism of action.

## 4. Materials and Methods

### 4.1. Plant Material

As a parental form, triticale cultivars bearing contrasting combinations of *DDW1* and *Grf3* alleles were chosen. Tall spring triticale cv. ‘Dublet’ with genotype *ddw1ddw1 Grf3(274)Grf3(274)* (Danko Hodowla Ro’slin Sp. z o.o., Poland) was used as the paternal form, and semi-dwarf winter triticale cv. ‘Hongor’ with genotype *Ddw1Ddw1 Grf3(262)Grf3(262)* (Lukyanenko National Grain Centre, Russia) was used as the maternal form. The hybrid F_1_ plants were grown in vegetation pots in 2016 in a greenhouse at the Center of Molecular Biotechnology. The F_2_ seeds were planted in vegetation pots at 10 seeds per pot. The F_2:3_ seeds of the spike of each of the F2 plants were manually threshed and combined into a single family. The homozygous F_2:3_ plants with Ddw1 and ddw1 alleles were selected by genotyping and grown to produce the F_3:4_ plants. The F_5_ and F_6_ seeds of the spike of each plant were obtained by mechanical threshing of F_4:5_ and F_5:6_ combining typical plants, respectively [35]. Each generation was additionally genotyped using molecular markers.

### 4.2. Field Experiment

A field experiment with plants was carried out in Moscow at the Field Experimental Station of the RSAU-Moscow Timiryazev Agricultural Academy (55°50′ N, 37°33′ E) in 2018–2019. Moscow (Central Non-Black Earth Region, moderate continental climate) is distinguished by a large amount of precipitation, moderate temperatures, and sod-podzolic soils. Sowing was performed in Moscow on May 5 in 2018 and on April 23 in 2019. Sowing in both years was carried out by the cassette method using an SKS-6-10 breeding seeder with the following parameters: plot length 1 m, in 4 rows with a row spacing of 30 cm and a distance between plots of 50 cm. Weeds were pulled out by hand, and the necessary treatment with pesticides was carried out to protect the plants from pests. Each plant was manually harvested upon reaching the full ripeness phase; the final harvest days were on 19 August 2018 and 23 August 2019 (Table 1).

In the present study, we used data from the analysis of F_4:5_ plant material obtained in 2018 (published in Kroupin et al. 2019) and newly obtained F_5:6_ plant material grown in 2019 [34]. The F_5_ and F_6_ seeds of the spike of each plant were obtained by mechanical threshing of F_4:5_ and F_5:6_ plants, respectively. The plants of F_4:5_ were grown and harvested in 2018 and mechanically threshed using an MKS-1M spike thresher (MZOK Company, Moscow, Russia). F_5_ seeds from each plant were combined into a single family (recombinant inbred line) and were used to grow F_5:6_ plants in 2019. The allelic states of *DDW1* and *Grf3* were determined in parental F_4:5_ (using stored DNA) and F_5:6_ plants such that the seeds from homozygous plants were selected for growing and subsequent analysis.

The weather conditions in 2018 and 2019 are displayed in Table 1. The numbers of plant samples in the analyzed families are shown in Table 2.

Analyzing the data presented in Table 1, it can be noted that in 2018, at the end of the growing season, unfavorable conditions occurred in the form of an extremely small amount of precipitation in August (25.6% of the norm), while 2019 had more even meteorological values, according to which the current climatic norm was approached.

### 4.3. Plant Structural Analysis

The following phenotype features of each plant were recorded (all spike parameters were measured in the main spike): plant height (PH, centimeters), spike length (SL, centimeters), spikelet number per spike (SNS), spike density (SD, calculated as tenfold SNS divided by SL), grain weight per spike (GWS, grams), grain number per spike (GNS), grain weight per spikelet (GNSpl, grams), thousand grain weight (TGW, calculated as thousandfold GWS divided by GNS, grams), number of fertile tillers (NFT), number of internodes (NI), heading date (HD, days after sowing), flowering date (FD, days after sowing), harvest index (HI), peduncle length (first internode below the spike, centimeters), second upper internode length (centimeters), third upper internode length (centimeters), fourth upper internode length (where present, centimeters), second lower internode length, and first lower internode (the lowest) length (centimeters). The harvest index was calculated as the ratio of the grain weight from the main spike to the total weight of the unthreshed spike and the peduncle. Flowering and heading phases were determined visually for the whole family. The seeds were counted by the SeedCounter application [51]. A total of 1293 spring triticale plants were analyzed in 2018, and 1193 were analyzed in 2019.

### 4.4. Plant Genotyping

**Figure 5:**
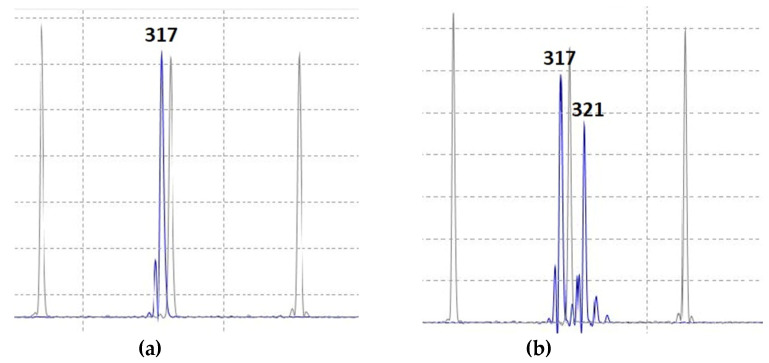
Identification of the allelic state of *DDW1* using fragment analysis of the REMS1218 marker amplicons: tall *ddw1* allele (**a**) and dwarfing *Ddw1* allele (**b**).

**Figure 6:**
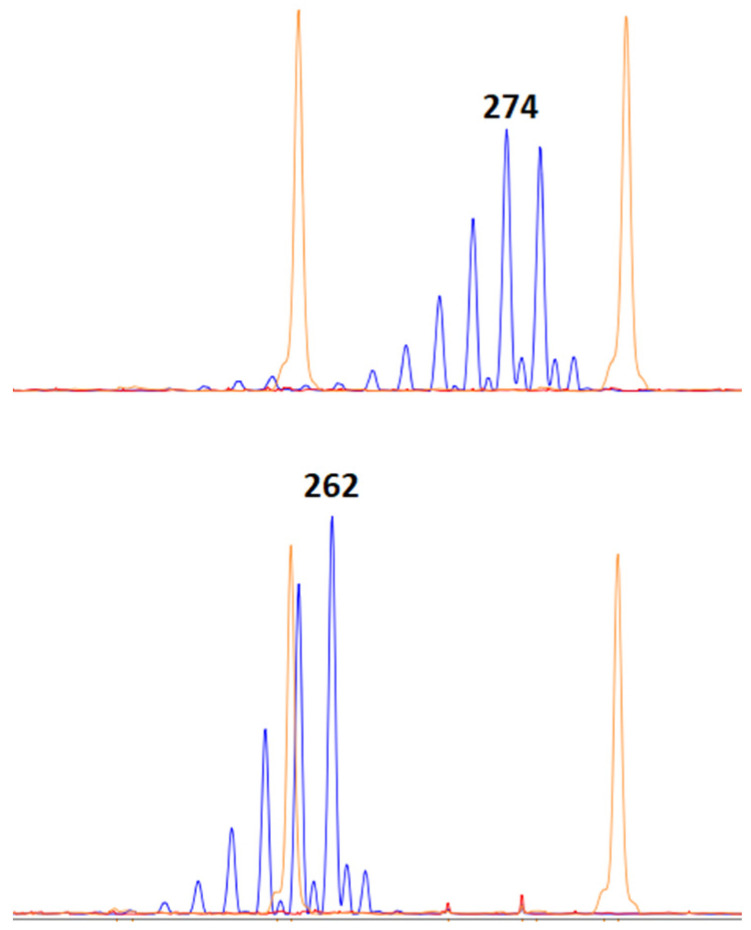
Identification of the allelic state of *Grf3* in parental spring triticale cultivars using fragment analysis: *Grf3*(274) in Dublet and *Grf3*(262) in Hongor.

During the tilling phase, a leaf fragment of approximately 2 cm long was sampled from each plant, and DNA was isolated by the CTAB method for verification of the *DDW1* and *Grf3* hybrid genotype. DNA was extracted from no fewer than two labeled plants per family to determine the allelic states of *DDW1* and *Grf3*. The primers targeted the microsatellite locus REMS1218, which is tightly linked to *DDW1*. The alleles of the REMS1218 microsatellites were assayed by PCR with subsequent fragment analysis performed using a Nanophor05 (Syntol, Russia Federation, Moscow) (Figure 5). Additionally, we used CAPS marker for the detection of the dwarfing *Ddw1* allele [52]. To detect polymorphism in the microsatellite locus of the 5’-untranslated region in *Grf3*, we designed primers F: 5’-GTAGGAGTAAAAGGCAAAAGCACG-3’ and R: 5’-ACAGGGAGGCAAAGGGCATC-3’ (Figure 6) [23,31]. The alleles of *Grf3* were assayed by PCR with subsequent fragment analysis performed using a 3130xl Genetic Analyzer (Applied Biosystems, Foster City, CA, USA).

### 4.5. Statistical Analysis

Data analysis was performed using R v3.6.3. An analysis of variation (ANOVA) between the allelic state of *Ddw1* and *Grf3* genes and phenotype was carried out using the Anova function from the “car” package [53] on a significance level of *p* < 0.05. For pair-wise comparisons, Tukey’s test was used. Plots were created using the “ggplot2” package [54].

## 5. Conclusions

In our study, we demonstrated a partial compensatory positive effect of the *TaGRF3*-2A gene on important yield components, such as grain weight of the main spike, thousand grain weight, and heading and anthesis time, in the presence of the dwarfing *Ddw1* allele. The *Ddw1* allele is necessary in modern triticale breeding, but it has a sharply negative effect on these traits. Based on this and our previous work, we propose an effective molecular marker for the allelic state of the *TaGRF3*-*2A* gene. This allows the *TaGRF3*-2A gene to be quickly incorporated into the breeding process to compensate for the negative effects of gibberellin-sensitive dwarfing genes.

## Figures and Tables

**Table 1:** Agroclimatic conditions of field experiments in 2018–2019.

Month	Sum of Precipitation (mm)		Average Temperature (°C)	
	2018	2019	2018	2019
May	44	58	16.1	16.2
June	54	55	17.2	19.6
July	85	64	20.3	16.7
August	20	48	19.8	16.4

**Table 2:** Numbers of analyzed F_4:5_ (2018) and F_5:6_ (2019) plant samples by genotype.

Genotype	F_4:5_(2018)	F_5:6_(2019)
*Ddw1Ddw1 Grf3*(274)*Grf3*(274)	45	268
*ddw1 ddw1 Grf3*(274)*Grf3*(274)	216	304
*ddw1 ddw1 Grf3*(262)*Grf3*(262)	351	472
*Ddw1Ddw1 Grf3*(262)*Grf3*(262)	106	262

## Supplementary Materials

The following are available online at https://www.mdpi.com/article/10.3390/plants10102012/s1, Table S1: Effects of *TaGRF3-2A* and *Ddw1* in the spring triticale ‘Hongor’ × ‘Dublet’ population F_4:5_ and F_5:6_ in the field experiments.
