# Supplementary material for: TaGRF3-2A Improves Some Agronomically Valuable Traits in Semi-Dwarf Spring Triticale
Source: Plants (Basel). 2021 Sep 25;10(10):2012. doi: 10.3390/plants10102012 (PMC8537337; doi:10.3390/plants10102012)
Supplement: Supplementary file 1 [file plants-10-02012-s001.zip › plants-1394084-supplementary.pdf]

Table S1. Effects of *TaGRF3-2A* and *Ddw1* in the spring triticale 'Hongor' × 'Dublet' population $F_{4:5}$ and $F_{5:6}$ in the field experiments.

Agronomic trait	$F_{4:5}$ (2018)				$F_{5:6}$ (2019)			
	Ddw1 *Grf3*(274)	*Ddw1* *Grf3*(262)	*ddw1* *Grf3*(274)	*ddw1* *Grf3*(262)	*Ddw1* *Grf3*(274)	*Ddw1* *Grf3*(262)	*ddw1* *Grf3*(274)	*ddw1* *Grf3*(262)
Plant height, cm	61.0 ± 5.4	51.9 ± 4.5	82.7 ± 5.0	81.3 ± 3.3	60.9 ± 3.1	54.0 ± 2.1	89.1 ± 5.9	85.7 ± 3.6
Spike length, cm	9.2 ± 1.2	8.1 ± 0.6	8.6 ± 0.5	8.7 ± 0.4	8.6 ± 0.2	7.7 ± 0.2	8.6 ± 0.3	8.5 ± 0.3
Spikelet number per spike	23.62 ± 3.14	21.83 ± 1.83	22.56 ± 1.21	22.94 ± 0.89	22.9 ± 0.67	22.02 ± 0.55	23.54 ± 0.7	23.22 ± 0.59
Spike density	25.35 ± 3.07	27.03 ± 1.81	25.7 ± 1.05	26.17 ± 0.89	26.46 ± 0.68	28.19 ± 0.68	27.46 ± 0.88	27.64 ± 0.79
Grain weight of the main spike, g	1.98 ± 0.44	1.33 ± 0.24	1.84 ± 0.25	2.04 ± 0.18	2.26 ± 0.19	1.98 ± 0.18	2.34 ± 0.21	2.43 ± 0.18
Grain number of the main spike	51.75 ± 10.9	43.0 ± 4.9	44.78 ± 4.52	48.88 ± 3.11	47.24 ± 3.33	44.31 ± 3.35	44.58 ± 3.67	46.26 ± 3.04
Grain number per spikelet	2.16 ± 0.22	1.95 ± 0.14	2.03 ± 0.17	2.14 ± 0.07	2.06 ± 0.11	2.03 ± 0.14	1.91 ± 0.13	2.01 ± 0.12
1000-grain weight, g	37.75 ± 2.78	31.81 ± 2.95	41.4 ± 2.9	41.41 ± 1.91	48.94 ± 2.34	45.98 ± 1.75	54.44 ± 2.77	52.28 ± 1.74
Vegetative mass of the main spike, g	2.53 ± 0.63	1.86 ± 0.31	2.40 ± 0.27	2.65 ± 0.22	2.93 ± 0.26	2.56 ± 0.19	3.04 ± 0.23	3.11 ± 0.21
Number of internodes	5.0 ± 0.0	5.06 ± 0.3	5.09 ± 0.14	5.16 ± 0.2	5.38 ± 0.23	5.24 ± 0.19	5.19 ± 0.16	5.47 ± 0.17
Heading time (days after sowing)	55.7 ± 3.7	61.5 ± 5.1	54.5 ± 0.6	55.0 ± 1.7	56.0 ± 1.2	63.1 ± 2.3	56.0 ± 1.4	56.9 ± 1.6
Flowering time (days after sowing)	59.2 ± 5.4	65.1 ± 5.5	57.0 ± 0.8	57.8 ± 1.9	58.9 ± 1.0	66.1 ± 2.7	59.1 ± 0.9	59.9 ± 1.4
Peduncle length, cm	20.9 ± 5.2	17.5 ± 1.8	30.4 ± 2.5	30.7 ± 1.7	20.9 ± 1.5	19.1 ± 1.0	31.6 ± 2.8	30.9 ± 1.6
2nd upper internode length, cm	14.2 ± 1.3	12.1 ± 1.6	20.5 ± 1.3	18.7 ± 1.1	12.8 ± 0.6	10.8 ± 0.6	19.9 ± 1.3	18.4 ± 0.9
3rd upper internode length, cm	8.0 ± 1.3	7.1 ± 0.9	11.9 ± 1.0	11.4 ± 0.5	8.5 ± 0.4	7.1 ± 0.4	13.7 ± 1.0	12.7 ± 0.6

2nd lower internode length, cm	5.0 ± 0.6	4.6 ± 0.6	7.6 ± 0.6	7.3 ± 0.5	5.2 ± 0.2	4.8 ± 0.3	8.2 ± 0.6	7.3 ± 0.5
1st lower internode length, cm	3.3 ± 0.7	2.2 ± 0.4	3.1 ± 0.3	3.2 ± 0.4	2.6 ± 0.3	2.7 ± 0.2	4.0 ± 0.5	3.8 ± 0.4
